# A case report of spontaneous pregnancy during hormonal replacement therapy for premature ovarian failure

**Published:** 2011

**Authors:** Firoozeh Akbari Asbagh, Mahbod Ebrahimi

**Affiliations:** Department of Obstetrics and Gynecology, Mirza Koochak Khan Hospital, Faculty of Medicine, Tehran University, Tehran, Iran.

**Keywords:** *Premature ovarian failure*, *Transient ovarian failure*, *Hypergonadotropic hypogonadism*, *Hypergonadotropic amenorrhea*, *Hormone replacement*

## Abstract

**Background:** Premature ovarian failure (POF) is a common condition; its incidence is estimated to be as great as 1 in 100 by the age of 40 years. Physiologic replacement of ovarian steroid hormones seems rational until the age of normal menopause. Temporary return of ovarian function and pregnancy may occur rarely in women with POF. We report a case of POF who conceived during hormone replacement therapy.

**Case**: A 30 years-old woman with confirmed POF after pelvic surgery and sever emotional stress conceived spontaneously.

**Conclusion:** Return of ovarian function and achievement of pregnancy is possible in women with POF.

## Introduction

 Premature ovarian failure (POF) is a mysterious disorder. It is defined by the association of amenorrhea, hypoestrogenism and elevated (menopausal) levels of serum gonadotropins before the age of 40 years (1). It is not a rare condition: its incidence is estimated to be as great as 1 in 100 by the age of 40 years, and 1 in 1000 by the age of 20 years (2). Although, the most cases of POF are idiopathic, with no identifiable etiology even after a thorough evaluation, diverse etiologies have been associated with POF: genetic aberrations, iatrogenic factors, autoimmune ovarian damage, infectious agents, toxins and environmental factors (3, 4). Netter *et al* have suggested that sever emotional stress could be cause of POF (5). Despite the absence of controlled evidence for this specific population, physiologic replacement of ovarian steroid hormones seems rational until the age of normal menopause (6, 7). This condition differs from normal menopause in several important ways. 

 Temporary return of ovarian function, as indicated by elevated estrdiol levels, follicle development, and even pregnancy may occur in women with idiopathic, iatrogenic or psychogenic ovarian failure (5-9). 

 Here, we report a case of POF who conceived during hormone replacement therapy.

## Case report

 A 30 years-old woman was referred to our infertility clinic, for evaluation of primary infertility with 7 years duration. She recalled experiencing thelarche at 11years of age and did not recall the timing of adrenarche. She had menarche at 12 years and reported regular menses, lasting 3to5 days. The significant points in her personal and past medical and family history were occasional migraine headaches without associated neurological deficits and mental retardation in her maternal uncle. Her physical examination revealed a healthy appearing woman with body mass index (BMI) of 26, Tanner stage V development, normal pelvic examination, including a well estrogenized vaginal epithelium. In this time, she was 25 years old. Routine infertility work-up including hormonal assay on 3^rd ^day of cycle (basal FSH level=6 IU/ L; LH level=5.5 IU/ L; E_2 _level= 27 pg/m L), semen analysis, hysterosalpingography, and transvaginal ultrasound revealed no abnormality with impression of unexplained infertility, controlled ovarian hyper stimulation (COH) and IUI was recommended. She conceived in the second cycle of COH and IUI. She had an ectopic gestation in ampullary portion of right tube that was treated with laparoscopic salpingectomy at the seventh week of gestational age. In laparoscopic view, uterus and left adnexa were unremarkable and right salpingectomy was performed by using electrocautery. No surgical complication occurred. The patient had one episode vaginal bleeding 4-5 weeks after operation. Then she experienced sever emotional stress (death in her family), and after this event, her menses ceased. Eight months later, she began experiencing hot flushes, dysparonia, and loss of libido. In this time, she was 26 years old. History and physical examination were unremarkable except for secondary amenorrhea with 8 months duration and hypoestrogenized vaginal epithelium. In vaginal smears, intermediate cells were seen. Transvaginal ultrsonography demonstrated normal size uterus with thin endometrum and small ovaries (right ovary 2.1cm^3^, left ovary 2.3cm^3^) with 3-4 primordial follicles in each ovary. Progesterone–withdrawal test was negative. Serum FSH and LH levels were high (FSH=62 IU/ L, LH =34.8IU/ L).Progesterone level was (0.3ng/m L), and estrdiol level was less than (10pg/ m L) .The3^rd ^day hormonal assay was repeated 4 months later (basal FSH level=135 IU/ L, LH level=88 IU/ L, E2 level= 10 pg/m L). CBC, ESR, FBS, serum creatinine, prolactin, androgen, ANA, Anti-ds ANA, U/A, and liver, thyroid, adrenal function tests were in normal ranges. Adrenal autoantibody tests were negative. DEXA study revealed mild osteopenia. Karyotype was 46 XX, fragile X mutation testing revealed normal size alleles with normal ranges of CGG repeats. Because of the unknown clinical value, serum anti ovarian antibody tests and ovarian biopsy did not request (9). She had serial hormonal (FSH, LH) assay. The last one belongs to 9 months prior to the recent pregnancy, which was showed high (menopausal) levels of gonadotropins. Hormone replacement as sequential regimen with 1.25mg of conjugated equine estrogen daily for 25days / month and 10 mg of medroxy progesterone acetate for 14days/ month was initiated. Daily weight bearing exercise, and calcium and vitamin D taking were advised. For infertility treatment, assisted conception with donated oocyte was suggested, but she did not accept this advice. Four years after starting sequential hormone replacement therapy, she noticed no return of vaginal bleeding for 6 weeks. At this time, her β-hCG level was positive, transvaginal ultrasonography showed an early intrauterine pregnancy. E_2_/progesterone replacement therapy was stopped. She is currently in second trimester (23weeks) of an uneventful pregnancy.

## Discussion

 Women with POF are not necessarily sterile; they have %5 chance of conceiving at some time after diagnosis (6). So the term POF is medically inaccurate. The terms ''hypergonadotropic hypogonadism'' and ‘‘premature ovarian insufficiency’’ are more accurate. However the most of spontaneous pregnancies occur while patients are receiving HRT, but this may not imply a cause-and- effect relation (7).

 Our patient had the pelvic surgery and emotional stress before spontaneous cessation of her menstruation. The effect of pelvic-adnexal surgery on ovarian function has been evaluated (10). Although no prospective studies of ovarian function and gonadotropin levels before and after pelvic-adnexal surgery have been done, some evidences indicate that such surgery sometimes affects ovarian function by compromising ovarian blood flow. Recovery after interventions that compromise ovarian blood supply would seem to be possible if sufficient collateral circulation develops and the resting follicles resume their cycles.

 Although, the mechanism involved in stress-induced ovarian failure is unknown, transient loss of ovarian function has been described in cases of major emotional stress (5). Netter *et al* reported hypergonadotropic hypogonadism secondary to emotional stress in 10 women 18 to 38 years of age. Only one of these patients had subsequent return of menses (5). While we cannot rule out idiopathic premature ovarian failure in our case, the sequence of events implies that the pelvic surgery and emotional stress influenced her ovarian function. 

 Although, we know that ovarian “failure” in POF does not mean permanent cessation of ovarian function, ‘but the likelihood of recovery of ovulation is not possible to predict POF (7).

 Although, different drug intervention such as various dosage of corticosteroids, estrogen, clomiphen, high-dose gonadotropin, recombinant FSH, danazol, and apoptatic inhibitors were recommended to induce ovulation in patients with POF, “ but the few randomized controlled trials that are available fail to demonstrate any significant improvement in ovulation and pregnancy rates(7, 9). Assisted conception (IVF) with donated oocyte was documented to be choice inthese patients (6,7). Advanced in technology of cryopreserved ovarian tissue transplantation and in-vitro maturation of oocytes derived from stem cells, may make it possible for some women with POF to use their own egg for IVF (11, 12,). The women with a significant family history of POF may consider oocyte or embryo cryopreservation since there are currently no entirely reliable tests to predict ovarian reserve (13, 14). 

 Pregnancy in the patients with POF is associated with significant fetal and maternal mortality and morbidity such as increased risk of a child with fragile X syndrome, intra uterine fetal death, pregnancy-induced hypertension, and postpartum adrenal crisis (15-17). So women who wish to avoid pregnancy should use a barrier method, because HRT or use of oral contraceptive pills will not prevent conception, perhaps due to the elevated gonadotropin levels in this condition (18).

## References

[1] Conway GS (2000). Premature ovarian failure. Br Med Bull.

[2] Luborsky JL, Meyer P, Sowers MF, Gold EB (2003). Premature menopause in a multi-ethnic population study of the menopause transition. Hum Reprod.

[3] Laml T, Schulz-Lobmeyr I, Obbruca A, Huber JC, Hartmann BW (2000). Premature ovarian failure: etiology and prospects. Gynecol Endocrinol.

[4] Pal L, Santoro N (2002). Premature ovarian failure (POF): discordance between somatic and reproductive aging. Res Rev.

[5] Netter A, Lambert A, Lumbroso P (1958). Etudes sur les amenorrhees: Les amenorrhees ovariplegiques. Bull Mem Soc Hop Paris.

[6] Panay N, Kalu E (2009). Management of premature ovarian failure. Best Practice and Research Clinical Obstet Gynecol.

[7] Goswami D, Conway GS (2005). Premature ovarian failure. Hum Reprod update.

[8] Van Kasteren YM (2001). Treatment concepts for premature ovarian failure. J Soc Gynecol Investig.

[9] Anassti JN (1998). Premature ovarian failure: an update. Fertil Steril.

[10] Sayegh R, Garcia CR (1992). Ovarian function after conservational ovarian surgery: a long-term follow-up study. Int J Gynecol Obstet.

[11] Donnez J, Squifflet J, Van Eyck AS (2008). Restoration of ovarian function in orthotopically transplanted cryopreserved ovarian tissue: a pilot experience. Reprod Biomed on line.

[12] Huang JY, Tulandi T, Holzer H (2008). Combining ovarian tissue crobanking with retrieval of immature oocytes followed by in vitro maturation and vitrification: an additional strategy of fertility preservation. Fertil Steril.

[13] Hefler L A, Grimm C, Bentz EK (2006). A model for predicting age at menopause in white women. Fertil Steril.

[14] Broekmans FJ, Kwee J, Hendriks DJ (2006). A systematic review of tests predicting ovarian reserve and IVF outcome. Hum Reprod update.

[15] Corrigan EC, Raygada MJ, Vanderhoof VH, Lawrence M, Neison LM (2005). A woman with spontaneous premature ovarian failure gives birth to a child with fragile X syndrome. Fertil Steril.

[16] Keegan DA, Krey LC, Noys N (2005). Younger (<35 years) donor egg recipients are at high risk for pregnancy- induced hypertention(PIH): A link between premature ovarian failure and PIH. Fertil Steril.

[17] Ambrosi B, Barbetta L, Morricone L (2003). Diagnosis and management of Addison,s disease during pregnancy. J Endocrinol Invest.

[18] Nelson L, Covington Sh, Rebar RW (2005). An update: Spontaneous premature ovarian failure is not an early menopause. Fertil Steril.

